# Combining Magnetic Resonance Spectroscopy and Magnetic Resonance Imaging in Diagnosing Focal Brain Lesions in Children

**DOI:** 10.7759/cureus.1541

**Published:** 2017-08-04

**Authors:** Farah Naz, Waseem A Mirza, Nauman Hashmani

**Affiliations:** 1 Fellow, The Aga Khan University; 2 Associate Professor, The Aga Khan University; 3 Ophthalmology, Hashmanis Hospital

**Keywords:** magnetic resonance imagining (mri), magnetic resonance spectroscopy, brain tumor

## Abstract

Introduction

We attempted to find the sensitivity and specificity of various pediatric brain masses in the Pakistani population while keeping histopathology or clinical diagnosis as the gold standard.

Methods

This was a retrospective study that was conducted from January 2007 to January 2016. We reviewed the records of 204 patients that presented to the radiology department of Aga Khan University Hospital (AKUH). Out of the 204, 135 pediatric patients in the 0–18 age group with focal brain lesions who underwent magnetic resonance spectroscopy (MRS) and a biopsy or clinical diagnosis were included. If histopathology was available, it was taken as the gold standard test; otherwise, clinical diagnosis was considered the gold standard.

Results

We had a total of 135 patients, of which 71 (52.6%) were male and 64 (47.4%) were female. The mean age represented was 7.2 ± 4.5 years with a range of 1–18 years. We found radiology (magnetic resonance imaging (MRI) and MRS) to have a 91.7% sensitivity and a 94.3% specificity for tumors. For leukodystrophy, there was a 64.3% sensitivity and a 97.3% specificity. On the other hand, infection and mitochondrial disorders had sensitivities of 35.7% and 21.7%, respectively, and specificities of 98.9% and 97.1%, respectively. The category labeled “others” had a sensitivity of 27.4% and a specificity of 86.0%.

Conclusion

A combination of MRI and MRS was highly sensitive and specific for tumors. For infections, leukodystrophy, mitochondrial disorders, and the category of “others,” it was highly specific but poorly sensitive.

## Introduction

 

Focal lesions and parenchymal changes in the brain are traditionally diagnosed by using neuroimaging studies, such as magnetic resonance imaging (MRI) or computed tomography (CT) scans. These scans provide morphological data only [1], which may carry a wide differential. Therefore, reaching a definitive diagnosis through MRI and CT scans alone was impossible [2]. For example, an intracranial abscess and a tumor can look anatomically identical [2]. To make this distinction, we relied on clinical judgment and an invasive histopathological diagnosis [2]. Magnetic resonance spectroscopy (MRS) can help differentiate between tumorous and nontumorous lesions by measuring biochemical markers in the brain [3]. It gives radiologists a tool to reach a definitive diagnosis noninvasively.

An MRS scan can help diagnose a wide variety of diseases such as tumors, cerebral ischemia, and trauma when used as an adjunct to the MRI [3]. It measures the resonant frequencies of various metabolites in the brain such as choline (Cho) and presents these values in a line graph with amplitudes [4]. These values themselves are not diagnostic; however, they must be interpreted along with the MRI [4]. Interestingly, MRS uses the same data produced by a conventional MRI; it only differs in the way it processes the data. Therefore, with the right scanner, this test is relatively easy to perform [4].

MRS is thought of as the future of neuroimaging [1] and has been found to be a reliable tool to diagnose brain tumors. Not only can it differentiate between intracranial lesions [5], but it can also aid in detecting the extent of the tumor in the nonenhancing areas of a mass [6]. Similarly, MRS can be used for both, making initial diagnosis and for guiding treatment options in the pediatric population [7]. This data has been widely studied in the western world but there is a paucity of data in South Asian countries. Similarly, there is no data, to our knowledge, in Pakistan regarding the accuracy of MRS and, therefore, we analyzed this data in our population. Furthermore, this study has one of the largest sample sizes in the world.

We wanted to study the sensitivity and specificity of MRS in differentiating pediatric brain masses in a large sample size in the Pakistani population while keeping histopathology or clinical diagnosis as the gold standard.

## Materials and methods

Study Design and Patients

This retrospective study from January 2007 to January 2016 included a total of 204 patients that presented to the radiology department of Aga Khan University Hospital (AKUH). Ethical approval was obtained from the Ethics Committee of AKUH. Out of the 204 patients, 135 pediatric patients in the 0–18 age group with focal brain lesions who underwent MRS and a biopsy or clinical diagnosis were included. If histopathology was available, it was taken as the gold standard test; otherwise, clinical diagnosis was used. Informed consent was obtained before performing MRS. We excluded all patients who did not have a radiologic diagnosis, a histopathological analysis, or a clinical record.

MRS Sequence

We used a single voxel method in our MRS examinations. Initially, we localized the lesion via a contrast MRI scan, and a voxel was subsequently placed at its location. Then, the water suppression method and the point-resolved spectroscopy (PRESS) method were utilized with an echo time (TE) of 135 and a response time (TR) of 1500.

We looked at the spectrum of N-acetyl aspartate (NAA), Cho, and creatinine (Cr). Additionally, we checked for the lipid or lactate peak on MRS. Ratios for Cho/Cr and Cho/NAA were calculated. NAA is the indicator of neuronal integrity with its highest point at 2.02 ppm. Cho, on the other hand, indicates cell turnover with its highest point at 3.22 ppm. Cr takes part in cellular metabolism with its peak occurring at 3 ppm. Normally, no lipid or lactate peak is observed in the brain and, even if present, they will overlap each other at 1.33 ppm [8].

Lesions can be differentiated based on the various ratios and peaks of these neurotransmitters. A raised Cho or decreased NAA combined with a rise in Cho/Cr and Cho/NAA ratios indicates a tumor. Additionally, lipid and lactate peaks are observed in high-grade neoplasms and infections. The Cho/Cr and Cho/NAA ratios are unchanged in nontumorous lesions [8].

First, we categorized MRS findings into two categories, normal and abnormal. Second, we categorized radiologic (MRS and MRI) and clinical diagnosis as normal, tumor, infection, leukodystrophy, mitochondrial disease, and others. Histology had the same categories but we omitted leukodystrophy and mitochondrial diseases, as we do not routinely perform biopsies for these conditions.

Statistical Analysis

All data was collected and input into SPSS version 16.0 (IBM, NY), and this was used to carry out all subsequent analyses. We used descriptive statistics to evaluate for sensitivity and specificity.

## Results

General Characteristics

We had a total of 135 patients, of which 71 (52.6%) were male and 64 (47.4%) were female. The mean age represented was 7.2 ± 4.5 years within a range of 1–18 years (Table 1).

**Table 1:** General Characteristics *Radiology is a combination of MRI and MRS

Variable	Male (n=71)	Female (n=64)	Total (n=135)
Age (Years)			
Mean			7.22
Standard Deviation			4.5
Range			1 - 18
Magnetic Resonance Spectroscopy (#)	71	64	135
Normal	39	27	66
Abnormal	32	37	69
Radiology* (#)	71	64	135
Normal	36	26	62
Tumor	5	12	17
Infection	3	3	6
Leukodystrophy	5	6	11
Mitochondrial Disease	5	3	8
Others	17	14	31
Histology (#)	11	14	25
Tumor	3	5	8
Infection	4	3	7
Others	4	6	10
Clinical (#)	69	61	130
Tumor	4	7	11
Infection	5	5	10
Leukodystrophy	6	8	14
Mitochondrial Disease	11	12	23
Others	43	29	72

Approximately half of the patients undergoing MRS (n=66) had normal scans. Similarly, when combining MRI with MRS, nearly half of the patients were normal as well (n=62). On radiologic diagnosis, defined as the combination of MRI and MRS, we found that 17 patients had tumors and the balance had a leukodystrophy (n=11), a mitochondrial disease (n=8), an infection (n=6), and other diseases (n=31).

When looking at histology, 8 patients had a tumor, 7 had an infection, and 10 were categorized as “others.” Clinically, approximately a fifth of patients had a mitochondrial disease (n=23), a tenth had a leukodystrophy (n=14), 11 had a tumor, 10 had an infection, and 72 were categorized as others.

Diagnostic Accuracy

We found radiology (MRS and MRI) to have a 91.7% sensitivity and a 94.3% specificity for tumors. For leukodystrophy, it had a 64.3% sensitivity and a 97.3% specificity. Conversely, infection and mitochondrial disorders had sensitivities of 35.7% and 21.7%, respectively, and specificities of 98.9% and 97.1%, respectively. The category labeled “others” had a sensitivity of 27.4% and a specificity of 86.0% (Table 2).

**Table 2:** Sensitivity and specificity of MRI and MRS

Diagnosis	Sensitivity (%)	Specificity (%)
Tumor	91.7	94.3
Infection	35.7	98.9
Leukodystrophy	64.3	97.3
Mitochondrial	21.7	97.1
Others	27.4	86.0

## Discussion

Traditional MRI and CT scans cannot reliably differentiate between tumorous and nontumorous lesions and, therefore, further investigations were required. MRS is a novel technique that can help distinguish these lesions by measuring the chemical composition of the brain. By using specific parameters and ratios, one can reliably determine the etiology of a particular condition without using invasive and painful procedures.

Using a combination of MRI and MRS, tumors could be reliably distinguished from non-neoplastic lesions with a sensitivity of 91.7% and a specificity of 94.3%. Other studies agree with our findings [8-10]. One study found MRS to be superior to an 8F-dihydroxyphenylalanine (F-DOPA) positron emission tomography (PET) scan in differentiating these two conditions [11]. Another study looked at differentiating tuberculomas from neoplastic lesions and concluded that a 3.8 ppm peak could reliably differentiate the two conditions [12].

Leukodystrophies yielded a moderate to low sensitivity (64.3%) and 97.3% specificity when using MRS as compared to clinical diagnosis. Our center does not routinely perform histopathologic studies for this diagnosis and, therefore, this was not available. While there are studies elucidating specific findings of leukodystrophies, the diagnostic accuracy of this method is yet to be reported. These findings include a raised Cho/Cr ratio with a suppressed NAA peak in Alexander’s leukodystrophy [13] and a lower concentration of all metabolites, except Cr, in adult onset autosomal dominant leukodystrophy [14]. Perhaps finding sensitivities and specificities for the various types of leukodystrophies would provide more accurate estimates but, unfortunately, this was not possible in our setup.

MRS had a poor sensitivity (35.7%) but a near-perfect specificity (98.9%) when studying leukodystrophies as compared to clinical diagnosis. As with leukodystrophy, no previous study has measured the diagnostic accuracy of MRS in these disorders. We did not have the data to elucidate individual infections, however. MRS is used extensively to study the human immuno deficiency (HIV) infection [15-16] and, in some studies, to study the hepatitis C virus (HCV) infection of the brain [17].

MRS proved to have poor sensitivity (21.7%) but highly accurate specificity (97.1%) for mitochondrial disorders. According to the Mitochondrial Medicine Society (MMS), various markers are available) for this diagnosis, including lactate, NAA, and NAA/Cr levels. In fact, NAA has been reported to be the best surrogate marker in the diagnosis of this disease [18]. Other studies agree with our assessment that MRS is not a sensitive tool to diagnose mitochondrial disorders: in one study, only 8 out of 29 showed lactate peaks [19] while in another, only 2 out of 11 did so [20]. In fact, in the first study, the same patient, when scanned twice, showed a normal MRS on one try and a lactate peak in the other [19].

Several limitations apply to this study. First, we could not determine the exact etiology of each disease. Second, this study was conducted in one center of a private hospital and, hence, a certain demographic was observed. Third, this was a retrospective analysis and, therefore, all associated limitations need to be taken into account.

## Conclusions

A combination of MRI and MRS was highly sensitive and specific for tumors. For infections, leukodystrophy, mitochondrial disorders and a category of “others” on the other hand, it was highly specific but poorly sensitive.  
